# Pentacyclic Triterpenoids from the Medicinal Herb, *Centella asiatica* (L.) Urban 

**DOI:** 10.3390/molecules14103922

**Published:** 2009-10-09

**Authors:** Jacinda T. James, Ian A. Dubery

**Affiliations:** Department of Biochemistry, University of Johannesburg, Auckland Park, South Africa; E-Mail: jacindaj@uj.ac.za (J.T.J.)

**Keywords:** *Centella asiatica*, asiatic acid, asiaticoside, centellosides, madecassic acid, madecassoside, pentacylic triterpenoids

## Abstract

*Centella asiatica* accumulates large quantities of pentacyclic triterpenoid saponins, collectively known as centelloids. These terpenoids include asiaticoside, centelloside, madecassoside, brahmoside, brahminoside, thankuniside, sceffoleoside, centellose, asiatic-, brahmic-, centellic- and madecassic acids. The triterpene saponins are common secondary plant metabolites and are synthesized *via* the isoprenoid pathway to produce a hydrophobic triterpenoid structure (aglycone) containing a hydrophilic sugar chain (glycone). The biological activity of saponins has been attributed to these characteristics. *In planta*, the *Centella* triterpenoids can be regarded as phytoanticipins due to their antimicrobial activities and protective role against attempted pathogen infections. Preparations of *C. asiatica* are used in traditional and alternative medicine due to the wide spectrum of pharmacological activities associated with these secondary metabolites. Here, the biosynthesis of the centelloid triterpenoids is reviewed; the range of metabolites found in *C. asiatica*, together with their known biological activities and the chemotype variation in the production of these metabolites due to growth conditions are summarized. These plant-derived pharmacologically active compounds have complex structures, making chemical synthesis an economically uncompetitive option. Production of secondary metabolites by cultured cells provides a particularly important benefit to manipulate and improve the production of desired compounds; thus biotechnological approaches to increase the concentrations of the metabolites are discussed.

## Introduction

*Centella* comprises some 50 species, inhabiting tropical and sub-tropical regions. This genus belongs to the plant family *Apiaceae (Umbelliferae*) and includes the most ubiquitous species *Centella asiatica*. This perennial creeper flourishes abundantly in moist areas and is a small, herbaceous annual plant of the subfamily Mackinlaya [1], previously included in Hydrocotyle [2], occurring in swampy areas of India, Sri Lanka, Madagascar, Africa, Australia [3], China, Indonesia, Malaysia, Australia and Southern and Central Africa [4]. The plant is clonally propagated by producing stolons that are characterized by long nodes and internodes which bear crowded cordate, obicular or reniform leaves and sessile flowers in simple umbels [5]. Depending on environmental conditions, the form and shape of the *C. asiatica* plant can differ greatly [6]. *C. asiatica,* also known as Gotu kola or Indian pennywort [7], is a medicinal plant that has probably been used since prehistoric times and has been reported to have been used for various medicinal and cosmetic purposes, thus becoming an important commercial product. This plant is listed as a drug in the Indian Herbal Pharmacopoeia, the German Homeopathic Pharmacopoeia (GHP), the European Pharmacopoeia, and the Pharmacopoeia of the People’s Republic of China [3]. According to World Health Organisation (WHO) monographs, Herbae Centellae should not contain less than 2% of the triterpene ester glycosides asiaticoside and madecassoside [8].

## Terpenoids as Natural Products and Secondary Metabolites

Secondary metabolites are natural products that often have an ecological role in regulating the interactions between plants and their environment. They can be defensive substances, such as phytoalexins and phytoanticipins, anti-feedants, attractants and pheromones [9]. The importance of plant secondary metabolites in medicine, agriculture and industry has led to numerous studies on the synthesis, biosynthesis and biological activity of these substances. It has been estimated that over 40% of medicines have their origins in these active natural products [10]. A prominent group of natural products are the terpenes and derivitized terpenoids.

## Chemical Diversity of Terpenoids

Several thousand terpenes and terpenoids occur in many genera of higher plants and organisms [11,12] and although often the structures of the various classes seem unrelated, detailed biochemical studies have revealed their biosynthesis patterns [13]. The terpenes are biosynthetically constructed from isoprene (2-methylbutadiene) units [10,14]. The C_5_H_8_ isoprenes polymerise and subsequently fix the number and position of the double bonds. The basic molecular formulae of terpenes are thus multiples of C_5_H_8._ Most terpenes have cyclic structures and are classified by the number of C_5_ isoprene units that they contain. Given the many ways the basic C_5_ units can be combined, it is not surprising to observe the amount and diversity of the structures [15]. The classes are: hemiterpenes consisting of a single C_5_ isoprene unit, monoterpenes (C_10_), sesquiterpenes (C_15_), diterpenes (C_20_), sesterterpenes (C_25_), triterpenes (C_30_), carotenoids (C_40_) and polyterpenes consisting of long chains of many isoprene units.

**Figure 1:**
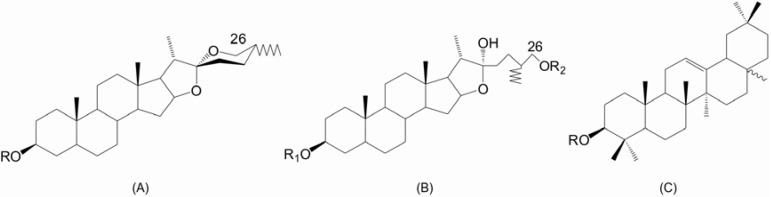
Aglycone skeletons of pentacyclic (A) steroidal *spirostane*, (B) steroidal *furostane* and (C) triterpenoid saponins. The R-group is a sugar moity [16]. Tetracyclic terpenes such as lanosterol, sitosterol and cycloartenol can also be derived from oxidosqualene through a different pathway utilizing cycloartenol synthase (CAS_1_) [19].

The triterpene group of compounds include sterols and triterpenes, which can accumulate as glycosides (saponins) in extensive amounts in plants [16]. Saponins are glycosylated (aglycone = sapogenin) secondary metabolites found in a variety of plant species [17]. Their surface-active properties are what distinguish these compounds from other glycosides [16]. Due to the fact that some of these saponins are the starting points for the semi-synthesis of steroidal drugs, these metabolites are highly sought after by the pharmaceutical industry [18]. Saponins are classified according to their aglycone skeleton. The first group consists of non-steroidal saponins, which are the most common and occur mainly in the dicotyledonous angiosperms. The second group consists of the steroidal saponins which are derived from the tetracyclic triterpenoids and isoprene units and are almost exclusively present in monocotyledonous angiosperms. Some claim a third class called steroidal amines, which are also referred to as steroidal alkaloids [7]. Steroidal saponins consist of a steroidal aglycone, a C_27_ *spirostane* skeleton which generally consists of a six-ring structure (Figure 1A). The hydroxyl-group in the 26-position may be engaged in a glycosidic linkage so that the aglycone structure remains pentacyclic (Figure 1B). This is referred to as a *furostane* skeleton. Triterpenoid saponins consist of a triterpene aglycone, which consists of a C_30_ skeleton, compromising a pentacyclic structure (Figure 1C).

## The Biosynthesis of *Centella* Triterpenes and Triterpenoids

**Figure 2:**
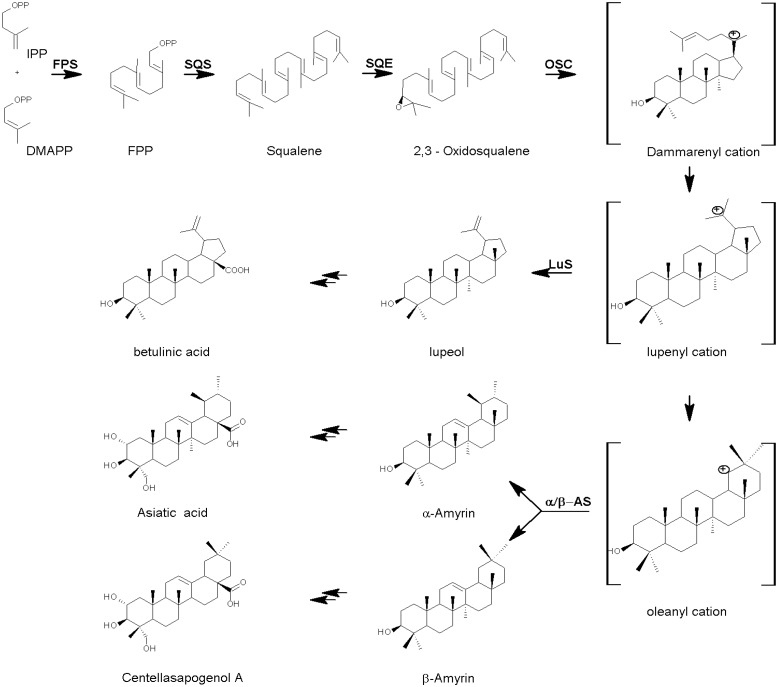
A simplified scheme of triterpenoid biosynthesis in *Centella*. Farnesyl diphosphate synthase (FPS) isomerizes isopentyl diphosphate (IPP) and dimethylallyl diphosphate (DMAPP) to farnesyl diphosphate (FPP), which squalene synthase (SQS) converts to squalene. Squalene epoxidase (SQE) oxidises squalene to 2,3-oxidosqualene. Oxidosqualene cyclase (OSC) enzymes cyclize 2,3-oxidosqualene through cationic intermediates (e.g. dammarenyl cation) to one or more cyclic triterpene skeletons. Other enzymes involved include α/β-amyrin synthases (α/β-AS) which can also form the lupenyl cation but further ring expansion and rearrangements are required before the deprotonation to α/β-amyrin, the precursors of the sapogenins, to generate the products listed in Table 1. Adapted from [19,20].

Terpene biosynthesis can be divided into four stages. Firstly, there is the formation of the isoprene unit isopentenyl diphosphate. There are two known major pathways for the biosynthesis of the isoprene unit, one based on mevalonic acid and the other one on 1-deoxyxylulose [10]. Secondly, there is the association of these units to form the (C_5_)_n_ isoprenoid backbone of the terpene families; thirdly there is the cyclization of these to generate the carbon skeletons. Finally, there are the interrelationships, hydroxylations and oxidations that lead to the individual terpenoids. The general biosynthesis of terpenes leading up to sterols has been reviewed extensively by Benveniste and others [10,20,21,22,23,24]. Triterpenes consist of six isoprene units and have the molecular formula C_30_H_48_. The linear triterpene squalene, is derived from the reductive coupling of two molecules of farnesyl pyrophosphate by squalene synthase (SQS). Squalene is then oxidized biosynthetically by squalene epoxidase (SQE) to generate 2,3-oxidosqualene. Oxidosqualene cyclases (OSCs) cyclize 2,3-oxidosqualene through cationic intermediates to triterpene alcohols or aldehydes including α- and β-amyrin and lupeol (Figure 2, [19,20]). 

These conversions are catalysed by OSCs, known collectively as triterpene synthases [21]. Plants biosynthesize diverse triterpenoids and their genomes encode multiple OSC enzymes to form these skeletons. The level at which the structural diversity of triterpenes is generated depends on the cyclization of 2,3-oxidosqualene by different OSCs such as cycloartenol synthase (CAS), lupeol synthase (LS) and α/β-amyrin synthase (AS) [25]. A phylogenetic tree analysis shows that OSCs have the same enzyme function from respective branches in the tree even though they were derived from different plants species. All triterpene synthases appear to have diverged from an ancestral CAS gene [26], but an independent origin for β-AS in eudicots and monocots has been proposed [19]. The triterpenoid cyclases are distinct from LS and CAS, and form discrete subgroups within the OSC superfamily [21].

Cyclization of 2,3-oxidosqualene through a *protosteryl* cation intermediate generates lanosterol and cycloartenol, the structural precursors to all the steroids, while cyclization through a *dammarenyl, baccharenyl* and *lupenyl* cation intermediates generates lupeol and α/β-amyrin [20], the precursors of the *Centella* pentacyclic triterpenoid saponins. The α/βAS enzymes cyclize oxidosqualene *via* the *dammarenyl* cation and allow further ring expansion and some rearrangement before deprotonation to α-amyrin and β-amyrin respectively [21]. α-Amyrin contains the *ursane* (C_19,_ C_20_ dimethyl) and β-amyrin the *oleanane* (C_20_ dimethyl) substitution patterns respectively.

Following cyclization, further diversity is conferred by modification of the products by oxidation, hydroxylation, glycosylation and other substitutions mediated by cytochrome P450-dependent monooxygenases, glycosyl transferases and other enzymes. Little is known about the enzymes required for these chemical elaborations. One common feature shared by all saponins is the presence of a sugar chain attached to the aglycone. Glycosylation is particularly important as the sugar chain is critical for the biological activity of many saponins [27,28]. The oligosaccharide chains are likely to be synthesized by sequential addition of single sugar residues to the aglycone, but little is known about triterpenoid glycosylation [21].

In the case of *C. asiatica*, the biochemical pathways involved in the synthesis of terpenes are active, as can be seen from the presence of monoterpenes and sesquiterpenes [29], and the well described pentacyclic triterpenes [2,30,31,32]. The synthesis of these compounds proceeds, as described above, from the cyclization of 2,3-oxidosqualene by a specific OSC, AS [25]. An AS, putatively involved in the synthesis of asiaticoside, has recently been described (CaβAS, GenBank accession AAS01523) [33]. 

**Figure 3:**
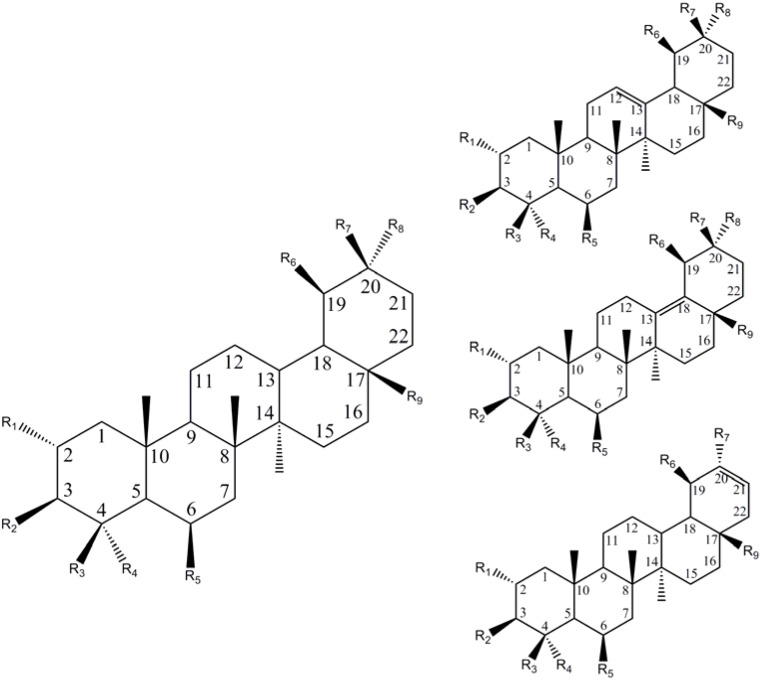
The model triterpenoid compound from *C. asiatica*. These triterpenes can occur in the *ursane* (R_6_, R_7_ = methyl) or *oleanane* (R_7_, R_8_ = methyl) types with double bonds occurring at C_12_-C_13_, C_13_-C_18_ or C_20_-C_21._

The basic structures of the *Centella* pentacyclic triterpenoid metabolites are represented in Figure 3. These can be divided according to the methyl substitution pattern on the C_19_ and C_20_ into the *oleanane* and *ursane* subtypes [34]. The most prominent of the *Centella* saponins are madecassoside and asiaticoside and their sapogenins (madecassic and asiatic acid). Other pentacyclic triterpenic acids and their respective glycosides which occur in *C. asiatica,* and reported in the older literature, include names like bramic -, madasiatic – [35], centic -, centoic -, centellinic -, centellic – [36], isodencentic acid, brahmoside, brahmioside [37], thankuniside, isothankuniside [38], and centelloside, amongst others. Structures of the pentacyclic triterpenes reported are compatible with the model scheme (Figure 3), with the exception of isothankunic -, centic - and centoic acids, that have an additional hydroxyl group attached to C_5_ [39]. A review of the literature has revealed duplicate names, synonyms and contradictory findings for the triterpenoid compounds of *C. asiatica*. Lack of structural data also hinders the assignment of names to structures. Isobrahmic acid, for example, was reported as identical to madecassic acid [38], with the latter being an isomer of terminolic acid [40]. Another report states that brahmic acid has been demonstrated to be identical to madecassic acid, while isobrahmic acid has been reported to be a mixture of asiatic - and madecassic acids [41]. The compounds brahmoside and brahminoside are recognised as sugar esters, similar to asiaticoside and madecassoside [41] but also containing arabinose. According to Dutta and Basu [42], thankunic and isothankunic acid are isomers of madecassic acid and the sugar-containing derivatives would be the glycosides thankuniside and isothankuniside respectively. *C. asiatica* plants from Sri Lanka were reported to contain centic, centoic, centellinic (the agylcone of centelloside) acids as well as indocentoic acid (the aglycone of indocentelloside) [43,44], but whether these names represent unique structures could not be ascertained. 

Table 1 summarizes a list of the *ursane* and *oleanane* centelloids where the compound name could be verified with a reported structure. Minor components such as the *lupaene* pentacyclic triterpene betulinic acid (3ß-hydroxy-20(29)-*lupaene*-28-oic acid), though structurally similar to the centelloids, are not included. 

**Table 1:** Structures of the pentacyclic triterpenes reported to occur in *C. asiatica* to date.

**R_1_**	**R_2_**	**R_3_**	**R_4_**	**R_5_**	**R_6_**	**R_7_**	**R_8_**	**R_9_**	**C=C**	**Name**	**Ref**
**Ursane (C_19_,C_20_ – dimethyl)**											
OH	OH	CH_3_	CH_2_OH	H	CH_3_	CH_3_	OH	COOH	-	2α,3ß,20,23-tetrahydroxy-urs-28-oic acid	[46]
OH	OH	CH_3_	CH_2_OH	H	CH_3_	CH_3_	H	COOH	20-21	2α,3ß,23-trihydroxy-urs-20-en-28-oic acid	[46]
OH	OH	CH_3_	CH_2_OH	H	CH_3_	CH_3_	H	COO-glc(1-6)glc(1-4)rha	20-21	Scheffuroside B	[46]
										2α,3ß,23-trihydroxy-urs-20-en-28-oic acid *O-*α-L-rhamnopyranosyl- (1-4)-*O*- β-D-glucopyranosyl-(1-6)-*O*- β-D-glucopyranosyl ester	[47]
OH	OH	CH_3_	CH_2_OH	H	CH_3_	CH_3_	H	COOH	12-13	Asiatic acid	[36,48]
										2α,3ß,23-trihydroxy-urs-12-en-28-oic acid	
OH	OH	CH_3_	CH_2_OH	H	CH_3_	CH_3_	H	COOCH_3_	12-13	Methyl asiatate	[48]
OH	OH	CH_3_	CH_2_OH	H	CH_3_	CH_3_	H	COO-glc(1-6)glc(1-4)rha	12-13	Asiaticoside	[34,35,49]
										2α,3ß,23-trihydroxy-urs-12-en-28-oic acid *O-*α-L-rhamnopyranosyl- (1-4)-*O*- ß-D-glucopyranosyl-(1-6)-*O*- ß-D-glucopyranosyl ester	
OH	-OH	CH_3_	CH_2_OAc	H	CH_3_	CH_3_	H	COO-glc(1-6)glc(1-4)rha	12-13	Asiaticoside C	[47]
OH	-OH	CH_3_	CH_3_	H	CH_3_	CH_3_	H	COO-glc(1-6)glc(1-4)rha	12-13	Asiaticoside D	[47]
OH	OH	CH_3_	CH_2_OH	H	CH_3_	CH_3_	H	COO-glc(1-6)glc	12-13	Asiaticoside E	[47]
H	OH	CH_3_	CH_2_OH	H	CH_3_	CH_3_	H	COO-glc(1-6)glc(1-4)rha	12-13	Asiaticoside F	[47]
OH	-OH	CH_3_	CH_2_OH	OH	CH_3_	CH_3_	H	COOH	12-13	Brahmic acid, Madecassic acid	[34,35,40,48,50]
										(6ß-hydroxy-asiatic acid)	
OH	OCH_3_	CH_3_	CH_2_OH	OH	CH_3_	CH_3_	H	COOCH_3_	12-13	Methyl brahmate	[48]
OH	OH	CH_3_	CH_2_OH	OH	CH_3_	CH_3_	H	CH_2_OH	12-13	Brahmol	[48]
OH	OH	CH_3_	CH_2_OH	OH	CH_3_	CH_3_	H	COO-glc(1-6)glc	12-13	Centellasaponin B	[34]
OH	OH	CH_3_	CH_2_OH	OH	CH_3_	CH_3_	H	COO-glc(1-6) glc(1-4)rha	12-13	Brahminoside	[34,35]
										Madecassoside	[51]
OH	OH	CH_3_	CH_3_	OH	CH_3_	CH_3_	H	COO-glc(1-6) glc(1-4)rha	12-13	Centellasaponin C	[34]
OH	O-L-Ara	CH_3_	CH_2_OH	OH	CH_3_	CH_3_	H	COOH	12-13	Arabinoside	[50]
										3-O-[α-L-ara]-2α,3ß,6ß,23-tetrahydroxy-urs-12-en-28-oic acid	
H	OH	H	CH_2_OH	OH	CH_3_	CH_3_	H	COOH	12-13	Isothankunic acid	[42]
										3α,5α,6ß,24-tetrahydroxy-urs-12-en-28-oic acid	
H	OH	H	CH_2_OH	OH	CH_3_	CH_3_	H	COO-glc(1-6) glc(1-4)rha	12-13	Isothankuniside	[38]
OH	OH	CH_3_	CH_3_	OH	CH_3_	CH_3_	H	COOH	12-13	Madasiatic acid	[34]
**Oleanane (C_20_, C_20_-dimethyl)**											
OH	OH	CH_3_	CH_2_OH	H	H	CH_3_	CH_3_	COOH	12-13	2α,3ß,23-trihydroxy-olean-12-en-28-oic acid	[35]
OH	OH	CH_3_	CH_2_OH	OH	H	CH_3_	CH_3_	COOH	12-13	Terminolic acid	[3,40]
OH	-OH	CH_3_	CH_2_OH	OH	H	CH_3_	CH_3_	COO-glc(1-6) glc(1-4)rha	12-13	Asiaticoside B	[3,50,34]
OH	-OH	CH_3_	CH_2_OH	H	H	CH_3_	CH_3_	-COOH	13-18	Centellasapogenol A	[35]
										2α,3ß,23-trihydroxy-olean-13-en-28-oic acid)	
OH	OH	CH_3_	CH_2_OH	H	H	CH_3_	CH_3_	COO-glc(1-6) glc(1-4)rha	13-18	Centellasaponin A	[34,35]
										Scheffoleoside A	
H	OH	CH_3_	CH_2_OH	OH	H	CH_3_	CH_3_	COOH	12-13	3ß,6ß,23-trihydroxy-olean-12-en-28-oic acid	[34]
H	OH	CH_3_	CH_2_OH	OH	H	CH_3_	CH_3_	COO-glc(1-6) glc(1-4)rha	13-18	Centellasaponin D	[34]

The biochemical pathways and genetic machinery required for the elaboration of this important family of plant secondary metabolites are still largely uncharacterized, despite the considerable commercial interest in this important group of natural products. This is likely to be due in part to the complexity of the molecules and the lack of pathway intermediates for biochemical studies.

## Biological Activities of *Centella* Triterpenoid Saponins and Sapogenins

*C. asiatica* synthesizes triterpenoid saponins as secondary metabolites as part of normal growth and development. Other chemical constituents that may contribute to the biological activities of *C. asiatica* may involve essential oils from this plant. Analyses of these oils have revealed monoterpenoids, oxygenated monoterpenoids, sesquiterpenoids, and oxygenated sesquiterpenoids with α-humulene, β-caryophyllene, bicyclogermacrene, germacrene B/D, myrcene, trans β-farnesene and *p*-cymol as the predominant constituents [29,52,53]. 

Interest in the centelloid molecules stems from their medicinal properties, antimicrobial activity, and their likely role as determinants of plant disease resistance [21]. Although classified as saponins, the saponin-like sugar esters of the triterpenoid acids exhibit low hemolytic activity [54].

The active constituents are well known for their clinical effects in the treatment of chronic venous diseases and wound healing disorders [55,56]. Many commercial formulations available contain madecassoside and asiaticoside in different ratios, depending on the source of the plant used to manufacture the final formulation (Table 2). Pharmacological studies performed have investigated the effects of undefined alcohol or aqueous extracts of *Centella*, as well as defined extracts. The following extracts are reported in the literature: TECA = titrated extract of *C. asiatica*, TTFCA = total triterpenoid fraction of *C. asiatica* and TTF = total triterpenoid fraction. TECA and TTFCA are combinations comprising asiatic acid (30%), madecassic acid (30%) and asiaticosides (40%) while TTF comprises asiatic acid and madecassic acid (60%) in combination with asiaticosides (40%) [2]. Some commercial products used in West Germany and France include Centasinum, Centelase, Emdecassol and Madecassol [2,54].

**Table 2:** Product range of extracts from *C. asiatica* indicating the specific chemical composition and treatment [8,57].

Extract	Chemical composition	Applications
Asiatic acid	>95% Asiatic acid	Anti-ageing cosmetics, application after laser therapy, cosmeceutics
Titrated Extract of *CentellaAsiatica* (TECA)	55-66% Genins	Anti-cellulite, slimming products, breast creams, stretch marks, scarred skin, anti-ageing cosmetics, moisturizing care
	34-44% Asiaticoside	
TECA cosmetics	>40% Genins	Anti-cellulite, slimming products, breast creams, stretch marks, scarred skin, anti-ageing cosmetics, moisturizing care
	> 36% Asiaticoside	
Heteroside	>55% Madecassoside	Slow release effect, anti-ageing cosmetics, for moisturizing night-creams
	>14% Asiaticoside	
Asiaticoside	>95% Asiaticoside	Anti-inflammatory, against irritated and reddened skin, anti-allergic
Genins	>25% Asiatic acid	Natural antibiotic, antibacterial properties, for anti-acne products, intimate hygiene
	>60% Madecassic acid	

In addition to the applications mentioned in Table 2, *Centella* extracts have been used for many ailments which have led to successful treatments (Table 3). Although none of the claims listed have been evaluated by the Food and Drug Administration (FDA), positive investigations have been done by various institutes and universities, which concluded that more research on the pharmacological and bio-medical activities of *C. asiatica* is called for. No recommended daily allowance (RDA) or dosage has been determined, but fresh leaves have been used in salads, or dried leaves used to make tea. Supplements are usually available in varying strengths and levels of purity. Crude preparations can cause allergic responses and nausea has been reported in cases of high levels of intake. A toxic dose of asiaticoside by intramuscular application to mice and rabbits was reported as 40-50 mg per kg body weight [58]. In oral applications, 1 g of asiaticoside per kg body weight has not proven toxic, and nearly all chemical trials have shown good tolerance by patients to extracts from *C. asiatica* or asiaticoside [58,59]. No cases of intolerance were observed following injections of Madecassol preparations [60] which is a *C. asiatica* extract comprising 40% asiaticoside, 29-30% asiatic acid and 1% madasiatic acid [2]. 

Although great progress has been made over the past decade in the study of biologically active components and the bioactivities of *C. asiatica*, the underlying mechanisms involving the physiological effects are poorly understood [5]. Most triterpenoid compounds in adaptogenic and medicinal plants are found as saponin glycosides. These sugars can be cleaved off in the gut by bacterial enzymes, allowing the aglycone triterpenoids to be absorbed. Uptake can be followed by insertion into cell membranes and modification of the composition. Membrane fluidity can be influenced to potentially affect signalling by many ligands and cofactors. In addition, the centelloids can potentially inhibit enzymes specifically or non-specifically. Literature supplies numerous examples of enzymes that can be inhibited by pentacyclic terpenoids, indicating the ability of these compounds to act broadly in a non-specific mode on multiple targets. The mode of inhibition of enzymes seems to be non-specific and based primarily on hydrophobic interaction with an enzyme's hydrophobic domain [75].

## Variation in Triterpene Production in *C. asiatica* Chemotypes

Natural products are an unsurpassed source of bioactive compounds and constitute a relevant economic resource for the pharmaceutical, cosmetic and food industry. Differences between varieties in medicinal plants of the same species (chemotypes) are common and variation in secondary metabolites has been observed with identical phenotypes and growth conditions, depending on plant origin [31]. Not surprisingly, significant differences in active constituents have therefore also been observed between samples of *C. asiatica* originating from different countries [76]. Moreover, the biosynthesis of major secondary metabolites is often either tissue or organ specific [31]. This also seems to be the case in *C. asiatica* where triterpenoid saponins, especially asiaticoside, could not be detected in undifferentiated cells of a Korean chemotype [30]. In contrast, detectable levels of the triterpenoids in cultured cells (callus and cell suspensions) were reported in South African chemotypes [32]. Asiaticoside biosynthesis seems to be concentrated in the leaves (0.4-1.4% dry weight) and the level of asiaticoside content is quite low in the roots of whole plants [30,33,77,78].

**Table 3:** Summary of the medicinal claims for *C. asiatica*. This table contains information on how *Centella* is used in alternative herbal treatments to treat various ailments and problems.

Medical claim					Description of treatment	Ref.
Skin ailments	Wound healing Treatment of skin disorders (such as eczema and psoriasis) Revitalising connective tissue Burn and scar treatment Cleaning up skin infections Leprosy Treatment of psoriasis				This tropical plant has been used in the Ayurvedic and traditional medicine in China, Malaysia and Madagascar, not only for wound healing but general well being as well as an anti-bacterial and anti-viral agent. Both the leaves and the entire plant can be used therapeutically. In traditional African medicine, it has been used for the treatment of leprosy. The asiaticoside content dissolves the waxy coat of the leprosy bacteria, thus allowing the bacteria to be destroyed by the immune system. *Centella* extracts are reported to be used topically in the adjunct treatment of surgical wounds and minor burns. **	[29,56] [61,62] [63] [64]
Circulation					Acts as a complementary treatment of ulcers of venous origin.	[56]
Arthritis and rheumatism					Extracts are taken orally to relieve symptoms of venous and lymphatic vessel insufficiency.	[7] [61]
Memory enhancement, vitality and longitivity.					In India, for the past 30 000 years of Ayurvedic medicine, it has been used from wound healing, a mild diuretic, increasing concentration and alertness, and well as for the treatment of anxiety and stress.	[56,63]
Cancer					In alternative health, this herb has been used to treat tumours and cancerous growths without suppressing the auto immune system or creating toxic wastes within the body. Cytotoxic and anti-tumour properties of the crude extract and in particularly purified fractions.	[56,65] [61]
A general health tonic, an aphrodisiac and immune booster					*Centella* assists in destroying toxic accumulation in the brain as well as in the nerves, while it helps to clear the body from heavy metals as well as drugs – including recreational drugs.	[61]
Respiratory ailments		Bronchitis Asthma				[56,63]
Detoxifying the body					Stimulates lipolysis and blood microcirculation and are thus used in the management of local adiposity or cellulite.	[56,66,61]
Slimming						
Diuretic						
Treatment of liver and kidneys					It has been used for centuries in the treatment of liver and kidney problems and has become a popular alternative treatment for people suffering from hepatitis and alcoholic liver disease. Managing diabetes.	[67,61] [29]
Sedative, Anti-stress, anti-anxiety and the treatment of depression					Ethanol extracts of roots had significant anti-stress activity. Activity against stress-induced gastric ulcer formation. Anxiolytic and sedative effects of the hydroalcoholic extracts of the leaves.	[67,68] [61] [63]
Antifungal properties						[63,69,^70^]
Insect anti-feedantMosquito repellent					Properties of isolated compounds of the extracts of rhizomes. Volatiles of *Centella.*	[71] [53]
Antibacterial activity				Periodontal disease Syphillis Hepatitis		[61,63,72,73,74]

In addition to the asiatic – and madecassic acids and their glycosides, other chemically diverse centelloid compounds as summarized in Table 1, have been isolated from *C. asiatica* and studied [34,76,79,80]. The reported composition of saponin mixtures of different sources of *C. asiatica* varies considerably (Table 4) as does the concentration of these compounds. The occurrence of these related triterpene ester glycosides and triterpene acids show that there are different varieties of *C. asiatica*, which can be summarized in Table 4 [36].

**Table 4:** Various saponins occur in *C. asiatica* due to the location and diverse environmental conditions [32,36,54].

Location / Source	Glycosides			Associated triterpene acids
	Saponin	Sapogenin	Sugar	
Madagascar South Africa	Asiaticoside Madecassoside	Asiatic acid Madecassic acid	Glucose and rhamnose	
Ceylon Sri Lanka	Centelloside	Centellic acid	Glucose and rhammose	Centic acid Centoic acid
India	Asiaticoside and Madecassoside	Asiatic acid Madecassic acid	Glucose and rhamnose	Brahmic acid
		Indocentoic acid		
	Asiaticoside Brahmoside Brahminoside	Asiatic acid Brahmic acid Brahmic acid	Glucose and rhamnose	Isobrahmic acid Betulinic acid
			Glucose, rhamnose and arabinose	
	Thankuniside	Thankunic acid	Glucose and rhamnose	Asiatic acid
	Isothankuniside	Isotankunic acid	Glucose and rhamnose	Asiatic acid

Gupta *et al*. [81] reported variable asiaticoside content in five lines of *C. asiatica* from India. Similarly, Rouillard-Guellec *et al*. [82] investigated the secondary metabolites in India and Madagascar, and reported that plants from the latter contained the highest level of asiaticoside. The distribution of asiaticoside and madecassoside throughout the plant was organ specific with leaves of both lines containing the higher content of these compounds. In a study of *C. asiatica* from Madagascar, asiaticoside content of between 2.6 and 6.42% dry weight was reported [83]. The authors achieved in vitro propagation of *C. asiatica* in a hormone free medium but these in vitro plants displayed lower asiaticoside content. Aziz *et al*. [31] reported two phenotypes of *C. asiatica* exhibiting differences in terpenoid content that were tissue specific and varied between glasshouse grown and tissue derived material. Triterpenoid saponin content was highest in leaves (asiaticoside and madecassoside concentrations of 0.7-0.9 and 1.1-1.6% dry weight were, respectively, reported), and roots contained the lowest content of asiaticoside. In their study, asiaticoside and madecassoside were undetectable in transformed roots and undifferentiated callus. Two morphologically distinct phenotypes of *C. asiatica* in South Africa were analysed in relation to the levels of triterpenoid saponins (madecassoside and asiaticoside) and their sapogenins (madecassic and asiatic acid), produced in undifferentiated cultured cells and leaves [32]. In both cases the triterpenoids present in undifferentiated cells (callus and cell suspensions) were lower compared to the levels in leaf tissues. The total content of the triterpenoids were generally comparable to that reported from India, Korea and Madagascar, but differences in the ratios of free acids to glycosides were observed. The reasons for this variability in the ratio between glycoside and aglycone can be due to climate, seasonal and geographical conditions, harvesting times and storage conditions [84].

Furthermore, the differences in the composition and type of triterpenoid molecules synthesized (Table 1) by various *C. asiatica* chemotypes can perhaps be attributed to genomic diversity and variation in the OSC and other genes involved in their biosynthesis [21,76,78], as well as the presence and activity of enzymes involved in the attachment of the sugar residues to the aglycones. Metabolic pathways for these triterpenoids should therefore be further investigated and the flux through these pathways elucidated to obtain a better understanding of the biochemical conversions that will allow the manipulation and exploitation of secondary product synthesis in *C. asiatica*.

## Manipulation of Centelloside Production in Cell and Tissue Culture

As in the case of most plant-derived pharmacologically active compounds, pentacyclic triterpenoids have complex structures, making chemical synthesis an economically uncompetitive option. Plant cell culture has been used in attempts to increase the production of bio-active secondary metabolites of pharmaceutical interest [85,86]. A particular important benefit is the potential ability to manipulate and improve the production of desired compounds within the plant cell through experimentation with cell culture. However, the relationship between cell differentiation and tissue organisation and the biosynthesis of secondary compounds is somewhat obscure. Secondary metabolite production may require interaction between roots and leaves with metabolic precursors generated in roots and passing to aerial parts of plants for bioconversion in leaves [85]. The biosynthesis of major secondary metabolites is often either tissue or organ specific [31], as found also in the case of *C. asiatica* triterpenoid saponins [87]. Plant secondary metabolites are normally synthesised by specialised cells, often at distinct stages of plant development and certain compounds are not synthesised, or synthesized at a low level, if cells remain undifferentiated as in cell suspensions [87]. The distribution of mRNA transcripts, enzymes and biosynthetic precursors within and between cells is an important component of regulation for secondary plant metabolic processes. In addition, many metabolic pathways are compartmentalised, enabling the separation of incompatible or competing reactions, and concentrating enzymes and metabolites [88].

One approach used to regulate metabolic pathways favouring the production of specific secondary metabolites has been to add precursors to the culture medium [89], though it is not known if this option has been investigated for enhanced production of triterpenoids in *C. asiatica* cells. The instability of cell cultures for the continued production of secondary products poses another problem; some cell lines lose the ability to synthesize the desired compound after prolonged culture.

In plant tissue cultures, stress induced by biotic and abiotic elicitors has been used to enhance production of biologically active secondary metabolites. It has been reported that fungal elicitation can lead to an overproduction of pentacyclic triterpenes in Tabernaemontana species instead of some other expected metabolites [90]. Another approach is to use plant-specific signal molecules such as methyl jasmonate (MeJa) to up-regulate key enzyme levels. It is known that exogenously applied MeJa can induce the biosynthesis of many secondary metabolites, including triterpenoid saponins [91]. The enzymes SQS and OCS were reported to be upregulated by MeJa treatment in cultured Glycyrrhiza glabra cells [91]. This upregulation was accompanied by enhanced concentrations of triterpenoid saponins. OCSs catalyze regulating steps in the isoprenoid pathway (Figure 2) and is responsible for the cyclization of 2,3-oxidosqualene, the common intermediate of both triterpene and phytosterol biosynthesis [21]. A significant increase in the asiaticoside levels of MeJa treated plantlets that were accompanied by a decrease in the content of free sterols were reported [25]. Previously, Kim et al. [33] reported an activation of ß-AS (an OSC) and a corresponding inhibition of expression of CS, responsible for the first step in sterol biosynthesis, in *C. asiatica* roots treated with MeJa. Thus, the inhibition of the branch point enzyme CS seems to result in increased flux through the triterpenoid pathway.

Biotechnological attempts to overproduce the quantities of asiaticoside through cell or tissue culture derived from a Korean chemotype have thus far encountered limited success [30,77,78]. Future studies to manipulate asiaticoside production, should be broadened to include all the triterpenoids obtainable from a specific chemotype in cultured plants and cells. 

The biosynthesis of the *Centella* triterpenoids can also be engineered by means of recombinant DNA technology along different steps of the pathways, once a particular rate-determining factor in a pathway has been identified. One approach to enhance terpenoid synthesis is to increase the flux of IPP and DMAPP by overexpression of their respective genes [92]. This potentially allows for the increased synthesis of all the triterpenes, but also for the manipulation of specific centellosides. Also, specific terpene synthases and OCSs may be modified or overexpressed to either regulate or enhance particular terpenoids [93]. In this regard, *C. asiatica* calli were cultivated in different media and the expression levels of the genes belonging to the biosynthetic pathway determined using RT-PCR [94].

## Conclusions

Due to its medicinal properties, interest in *C. asiatica* has increased over the years and there have been studies on the enhanced production of these centellosides as well as cloning of genes in their biosynthetic pathway. The production of these compounds and expression in differentiated (leaves and roots) and non-differentiated (calli) cells have been investigated. Metabolic pathways for these triterpenoids should be elucidated to obtain a better understanding of the biochemical conversions that will allow the manipulation and exploitation of secondary product synthesis in *C. asiatica*. There is a need for additional studies to be done to evaluate the genetic resources of the plant for variation in growth, morphology, and yield related characteristics which can, in turn, be utilized to identify high yielding populations suitable for agronomical and plant breeding programs.
